# Supplementary material for: Qualitative study of the consequences of vaccine-induced immune thrombocytopenia and thrombosis: the experiences of family members
Source: BMJ Open. 2023 Dec 9;13(12):e080363. doi: 10.1136/bmjopen-2023-080363 (PMC10729125; doi:10.1136/bmjopen-2023-080363)
Supplement: Supplementary data [file bmjopen-2023-080363supp001.pdf]

Appendix 1:

Topic guide

Record!

Check consent - aware that is being recorded, can drop out at any time, audio recordings will be destroyed and anonymized data used in research outputs.

Age

Gender

Relationship to VITT person

Want to discuss the impact of VITT on you and perhaps any others within the family affected by this… start with what has happened and then its impact on you/family

1. Describe the family

Version 1 24/6/2022[Document title]

2. Thinking back to the time when partner/family member developed VITT, how did this impact on you and other members of the family?

   Prompts include…

   - what experiences for you/them during initial onset of symptoms/hospitalization?
   - When did you find symptoms were VITT?
   - What about family? How broke the news, how took it?

3.What were the psychological consequences of these experiences at the time for you/them?

Prompts include..

- Impact of isolation due to covid
- Fear, worry

3. What physical/psychological symptoms has person with VITT since then? How have these impacted on your (joint) life?

   Prompts include…

   - Day to day – mobility, pain, fatigue restrictions
   - Longer term – finance, job
   - Impact on family

4. What were the longer-term psychological consequences of living with a person with VITT?

Prompts include

- Health anxiety
- Depression
- Concern for partner
- Anger/frustration
- Restricted family quality of life

5. If partner deceased.. what have been your experiences since your loss?
   Prompts include
   - Emotional
   - Contact with other bereaved people
   - The system.. hospital, VDPS etc
   - How impacted on family

6. *If time*, what are views on vaccination/covid now?

Thanks for participating. Reminder that if need support get in touch with Thrombosis UK on email in the PIS.

Version 1 24/6/2022[Document title]

Will send copy of findings…
