# Supplementary material for: Qualitative study of the consequences of vaccine-induced immune thrombocytopenia and thrombosis: the experiences of family members
Source: BMJ Open. 2023 Dec 9;13(12):e080363. doi: 10.1136/bmjopen-2023-080363 (PMC10729125; doi:10.1136/bmjopen-2023-080363)
Supplement: Author's manuscript [file bmjopen-2023-080363.draft_revisions.pdf]

BMJ Open is committed to open peer review. As part of this commitment we make the peer review history of every article we publish publicly available.

When an article is published we post the peer reviewers' comments and the authors' responses online. We also post the versions of the paper that were used during peer review. These are the versions that the peer review comments apply to.

The versions of the paper that follow are the versions that were submitted during the peer review process. They are not the versions of record or the final published versions. They should not be cited or distributed as the published version of this manuscript.

BMJ Open is an open access journal and the full, final, typeset and author-corrected version of record of the manuscript is available on our site with no access controls, subscription charges or pay-per-view fees (http://bmjopen.bmj.com).

If you have any questions on BMJ Open's open peer review process please email info.bmjopen@bmj.com

BMJ Open

A qualitative study of the consequences of vaccine-induced immune thrombocytopenia and thrombosis: the experiences of family members.

Journal:	*BMJ Open*
Manuscript ID	bmjopen-2023-080363
Article Type:	Original research
Date Submitted by the Author:	28-Sep-2023

Complete List of Authors:	Bennett, Paul; Swansea University, Psychology<br>Celik, Filiz; Swansea University<br>Winstanley, Jenna; No affiliation<br>Hunt, Beverley; Kings Healthcare Partners, Thrombosis and Haemophilia Centre<br>Pavord, Sue; Oxford University Hospitals NHS Foundation Trust,

Keywords:	Bleeding disorders & coagulopathies < HAEMATOLOGY, PUBLIC HEALTH, QUALITATIVE RESEARCH

SCHOLARONE™ Manuscripts

I, the Submitting Author has the right to grant and does grant on behalf of all authors of the Work (as defined in the below author licence), an exclusive licence and/or a non-exclusive licence for contributions from authors who are: i) UK Crown employees; ii) where BMJ has agreed a CC-BY licence shall apply, and/or iii) in accordance with the terms applicable for US Federal Government officers or employees acting as part of their official duties; on a worldwide, perpetual, irrevocable, royalty-free basis to BMJ Publishing Group Ltd ("BMJ") its licensees and where the relevant Journal is co-owned by BMJ to the co-owners of the Journal, to publish the Work in this journal and any other BMJ products and to exploit all rights, as set out in our licence.

The Submitting Author accepts and understands that any supply made under these terms is made by BMJ to the Submitting Author unless you are acting as an employee on behalf of your employer or a postgraduate student of an affiliated institution which is paying any applicable article publishing charge ("APC") for Open Access articles. Where the Submitting Author wishes to make the Work available on an Open Access basis (and intends to pay the relevant APC), the terms of reuse of such Open Access shall be governed by a Creative Commons licence – details of these licences and which Creative Commons licence will apply to this Work are set out in our licence referred to above.

Other than as permitted in any relevant BMJ Author's Self Archiving Policies, I confirm this Work has not been accepted for publication elsewhere, is not being considered for publication elsewhere and does not duplicate material already published. I confirm all authors consent to publication of this Work and authorise the granting of this licence.

For peer review only

A qualitative study of the consequences of vaccine-induced immune thrombocytopenia and thrombosis: the experiences of family members.

Paul Bennett PhD (corresponding author)
Thrombosis UK and Swansea University
School of Psychology
Swansea University
Swansea
SA2 8PP
p.d.bennett@swansea.ac.uk

Filiz Celik PhD
School of Psychology
Swansea University
Swansea
UK
Filiz.celik@swansea.ac.uk

Jenna Winstanley BSc
No affiliation
jvwinstanley@outlook.com

Beverley J Hunt OBE
Thrombosis & Haemophilia Centre
Kings Healthcare Partners
London
Beverley.hunt@gstt.nhs.uk

Sue Pavord FRCPath
Department of Haematology
Oxford University Hospitals NHS FT
Sue.pavord@ouh.nhs.uk

For peer review only

ABSTRACT

Objectives: To explore the experiences of family members of patients who died or survived following a diagnosis of Vaccine-induced Immune Thrombocytopenia and Thrombosis (VITT).

Design: A semi-structured qualitative study, conducted via Zoom.

Setting: Participants discussed their experiences during hospitalisation and following discharge.

Participants: Sixteen family members of VITT patients (survivors = 11; bereaved = 5), recruited via a Facebook support group and advertising on Twitter.

Results: Analysis identified two themes common to both groups of participants: the stress of hospitalisation, and the experience of multiple losses. A third theme, living with VITT, was unique to the survivor group, and a fourth, battling against the system, was predominantly reported by bereaved participants.

Conclusions: This is a significantly challenged group of people, with multiple emotional, financial, social and psychological losses. These losses have been compounded by experiences of limited governmental and societal recognition of the problems they face.

Key words: family, vaccine-induced immune thrombocytopenia and thrombosis, VITT, psychosocial sequelae

STRENGTHS AND LIMITATIONS OF THIS STUDY

Strengths

- The first study to explore the impact on the families of patients surviving and dying of VITT
- The sample included approximately 10 per cent of the surviving relatives of all UK patients who died from VITT.

Limitations

- Although attempts were made to widen recruitment, the majority were from a VITT support group provided by Thrombosis UK and in response to tweets via Twitter (X)

Word count: 4823

For peer review only

INTRODUCTION

We[1] have previously explored the experiences of patients diagnosed with Vaccine-induced Immune Thrombocytopenia and Thrombosis (VITT) over the 18 months since their acute presentation[1]. During these interviews, it became apparent that the experiences of patients' families (including health, financial, and social concerns) largely mirrored those of the patients, although this was not formally explored in the study. Furthermore, a mortality rate of more than 20 per cent[2], necessarily meant that some families had to cope with the sudden and unexpected death of a family member, often at a young age. The present study aimed to explore the experiences of both bereaved and survivor families.

METHOD

Qualitative interviews were conducted 15-24 months following a diagnosis of VITT.

Patient and public involvement

The study protocol was discussed separately with several relatives of patients with VITT prior to conducting the research. In addition, all participants had repeated sight of the paper as it developed to ensure their agreement with the themes identified, quotations cited, and degree of anonymity provided.

Recruitment and sample

Participants were family members of patients with a confirmed diagnosis of VITT, recruited via social media: (i) a closed VITT Facebook support group hosted by Thrombosis UK, (ii) the Thrombosis UK Twitter feed, and (iii) re-tweets by individuals known to members of the Facebook group or who read the Twitter feed. The final sample comprised 16 participants. Eleven were family members of surviving VITT patients (nine partners, two adult daughters). Five were bereaved. There were 13 women and 3 men, with a man age of 46 years (range 21-59).

Method

Interviews were conducted between August 2022 and July 2023. People who contacted the study team were sent a Participant Information Sheet and confirmed their willingness to participate by making further contact with the study lead. After providing informed consent, participants took part in a semi-structured virtual interview on Zoom, addressing their experiences of hospitalisation and the longer-term impact of living with a VITT patient or of

For peer review only

For peer review only - http://bmjopen.bmj.com/site/about/guidelines.xhtml

their bereavement (see appendix 1). Three people (PB, FC, JW,) trained in clinical/counselling psychology and with previous experience of VITT[1], conducted the interviews.

Data Analysis

Interviews were recorded on Zoom and a 'clean' transcript determined, after which the original recordings were deleted. All identifying elements were removed from the transcripts, which were analysed by PB using inductive thematic analysis[3]. Audio recordings were listened to repeatedly to identify relevant initial codes and textual units. Extracts and phrases were used to identify potential themes, with relevant quotes gathered within identified themes. Themes and sub-themes derived from the data were checked and validated by FC; any differences were resolved through discussion.

RESULTS

Of the themes identified, theme 1, the stress of hospitalisation, has commonalities across both groups of participants. Theme 2, Living with VITT, is unique to families of survivors. Theme 3, multiple losses, has commonalities across both groups. Theme 4, battling against the system, is predominantly reported by bereaved participants. Table 1 provides an overview of the themes and sub-themes.

Table 1: Key themes and sub-themes

Theme	Sub-theme
1. The stress of hospitalisation	The dawning light of seriousness
	Breaking the bad news
	Waiting for news
2. Living with VITT	Coming home is not the end
	The whole family has to adjust
3. Multiple losses and some gains	Loss of a loved one
	Loss of a future
	From trauma, growth
4. Battling against the system	

Theme 1: the stress of hospitalisation

For peer review only - http://bmjopen.bmj.com/site/about/guidelines.xhtml

The dawning light of seriousness

Most participants were concerned at the initial onset of symptoms. However, this anxiety increased significantly when participants became aware of the severity of their family member's diagnosis, often given over the phone due to Covid pandemic imposed visiting limitations. Anxiety was frequently exacerbated by incomplete understandings of the medical condition:

> *I had a consultant phone, and she was saying, … She's reading through a big, long list of things. …. She's going down this list. And then she says, "Oh, and she's had a stroke." That's the one word that my mind picked up on because that's what I knew about. … She went, "Yes, but don't worry about that. That's the minor thing what's wrong with her." That's when you think, God … The stroke is the minor thing! … what's the rest of it!? (*5: survivor*)*

Many relatives of survivors were fearful they would die. As one participant noted, '*I suddenly woke up, like bang at four o'clock in the morning, and I thought… she could actually be dead now. She could be dead now.'* (7: survivor). For those who did die, there was little or no time for preparation. Four of the five participants were rung at home shortly after admission, told that exploratory surgery have proven unsuccessful, and that their partner had very limited time to live:

> *I remember crying a hell of a lot. But because it was just so quick... I was more shocked. I remember physically feeling sick to my stomach, and then it just sort of clicked… scared sick or something or something like that because I was literally physically sick. I just remember feeling really raw and empty, and just shocked.* (4: bereaved)

> *And I was with him then. His parents rang my mobile while I was with him... and they heard me tell him that they loved him... And then they hung up.* (3: bereaved).

Children's worries about their parent's health manifested in several ways. One parent (5: survivor) had to sleep with their child to help keep them calm. One child resorted to prayer (*You know how children pray by the bedside... and she was going. Daddy, please don't die, please don't die* 6: survivor*)*, while others acted out their stress at school (*A teacher contacted me and said, "Your son's been in a bit of a mess in the class because he's worried about his mom".* 2: survivor).

For some people, trauma continued in the form of flashbacks or memories of critical events for many months: *I think now it's hitting me. Because I'm getting flashbacks and things like that. … And my mind's trying to deal with it. … And just sort a lying there, just shaking and not knowing*

For peer review only - http://bmjopen.bmj.com/site/about/guidelines.xhtml

who I am or where I am; and I'm not recognizing my room and not knowing my name. (1: survivor).

Breaking the bad news

In the face of their own concerns, participants had to determine when and how to inform others of their partner's condition. Telling adult family members was challenging (*You know, like you get your stress headaches … and the pressure and the stress of how to put it…* 1: survivor). Telling children was perhaps even more challenging. Participants had to impart that their parent was sufficiently ill to require hospitalisation, but try to maintain optimism and confidence (despite their own reservations) that they would return home:

> *I was thinking, what do I say? Do I tell them? I thought it was probably best not to at this stage because they will not sleep… it'll just make them in a bit of a mess…. I was like, "Oh, well, she's staying in overnight, and everything will be all right, let's get to bed." The other thing I thought about it was, what if I'm telling the kids, "Everything's all right, everything's all right.", then in the morning, I'm going to get this message saying, your wife had a stroke or died in the night, and then I'm going to tell them this and actually she wasn't all right, and I was lying to them.* (2: survivor)

Waiting for news

Following admission to hospital, anxieties were often exacerbated by Covid-related visiting restrictions which resulted in very limited contact with clinicians. Relatives knew their condition was serious, but most struggled to track how their relative was progressing:

> *.. The biggest thing was not being with him. That was the hardest thing, and just waiting for... Because the hospital, they never rang me. The nurses, the doctors never rang me to say, "This is what's happened."* (5: bereaved)

When achieved, communication could also be challenging and distressing for both participants and health professionals:

> *… because they took all of the hope away from me. One particular doctor told me over the phone that all of the interventions they were doing, pretty much, were a waste of time and never called me back to talk me through that because I had to put the phone down.* (8: survivor)

For peer review only

Of the few that did get to see their relative, some were ‘sneaked in’. Others fought to achieve access:

> *Care for the patient's family was just non-existent at the time because it was locked down. It was covid…I think that added to everyone's trauma. I was lucky because I had to fight for it … but that shouldn't have been the case. They should have applied some sense of logic and allowed me to put the risk that I'd rather be with him if he died than not.* (8: survivor)

Communication with patients often involved the use of social media, which could be reassuring, but could also be stressful:

> *I just laid here just waiting for the phone to go. Just waiting for a message saying, ‘I'm fine’, you know. He was losing all track of time and he'd send me messages at like 4:00 o'clock in the morning... And I'm like, I'm trying to sleep here, but I kept it on because obviously … It was just horrendous. All the things that go through your head. Just staring at the four walls and. Yeah, just waiting.* (3: survivor)

The lack of direct contact with clinicians and patients led to anxieties about the veracity of patient reports about their health:

> *I think there was always a worry that my mum was not going to be entirely 100% honest with it, not out of anything other than just like trying not to worry me. I found it hard…* (7: survivor)

Finally, participants worried about the psychological impact of hospitalisation on the patient as well as experiencing their own stress:

> *Whenever she's been ill or anything like that, I've always been with her. Not to be with her is quite upsetting because I'm then thinking of how scared and upset she's going to be… I came home and literally stood in the garden and cried. That's being military, 23 years in the army, quite hands-on, quite a person to get things done and fix things and make things right. It's horrible not being in control.* (9: survivor)

Theme 2: Living with VITT

Coming home is not the end

For peer review only - http://bmjopen.bmj.com/site/about/guidelines.xhtml

For survivors, initial relief following discharge was frequently replaced by the significant challenges of living with, and caring for, a very sick individual, who may have changed significantly from the person before admission to hospital.

> *And the mood swings. You know, I think it's 'cos it's coming down off all the steroids and everything else. The mood swings that he had was... awful … just horrendous. He wasn't aggressive, but he just didn't realise how snappy he was getting…You just, you didn't know whether to speak, not speak. Do you go in? Do you not? You know, it was horrible. I remember my girl crying herself to sleep in my arms because it wasn't her dad that came home.* (3: survivor)

Participants were concerned about the risk of future problems, and how to avoid them. They expressed concerns about their partner's health as well as awareness that medical aid was not immediately available:

> *Yeah. I suppose when she came home, I was treating her with kid gloves. I felt like she was a bit, she was super-delicate, and I kept thinking don't bang your head, don't do anything when you knock your head or… And that probably got on her nerves a bit…* (2: survivor)

For some, recovery from VITT meant a slow acceptance of improving health, confidence that they will not experience further health crises, and shifts towards a more normal life. For those nine individuals with continuing symptoms, the stresses and strains continued.

> *I lie there at night listening to his breathing 'cos …. he struggles a lot in the night. And some nights I nudge him to make sure he's going to take the next breath, cause he's really struggling to breathe, so I know if in the night his breathing is real bad I know in the morning, what kind of day it's gonna be.* (3: survivor)

As well as the daily demands of caring, participants noted the toll of repeated hospital appointments (one participant noted their partner's 250th appointment had recently passed) either for monitoring, rehabilitation, or continuing investigations. Others reported the sheer stress of caring:

> *…. in the early days, it was a massively dark time in the fact that she was so angry and upset. She didn't want to be here anymore. Trying to keep her going, trying to keep her functioning, trying to get the right pills in her, trying to work out what's going on and trying to work out what appointment's coming next. … But we still have other days where*

For peer review only - http://bmjopen.bmj.com/site/about/guidelines.xhtml

it's dark and she gets angry and upset and she doesn't want to be here anymore. So, dealing with that is hard work... (9: survivor)

I went through a really tired stage where I had this like chronic fatigue and I just, and it would just hit me wherever I was, and I'd actually have to go and lie down no matter what time of day it was. So that's exhaustion and fatigue. (1: survivor)

Most families reported little or no systematic support following discharge (although some individual clinicians were highly supportive) even when the patient had experienced significant life-changing conditions:

..but there seems to be no sort of holistic approach to supporting families when a loved one has been through a life-changing condition in the hospital or in a coma. There's no pathway for that to support us. They just look at the patient... (8: survivor)

The whole family has to adjust

The impact of the illness frequently affected the whole family. For some, as noted above, this involved daily commitments to care on a 24-hours a day basis. For others, the commitment may have been less, but life plans were nevertheless changed.

My husband's job is in London, and we were thinking of moving up towards London. And after this happened, I decided let's buy a house here in X so I'm just three minutes away from my mum. And then my brother bought his and he's two minutes away from me. So, we're all literally in a line. So, we're all around to be here for her. (11: survivor)

At times, more subtle issues affected the whole family. Two participants, for example, reported the need to adjust the family day to day noise, and for their partner to have periods of quiet:

... we can't behave the way we used to. Like before, we'd carry on as a normal day to day family messing around, noise, you know, you'd have all that ... And now it's like you're kind of walking on egg shells all the time because you don't know.... (6: survivor)

Another reported the adjustments necessary due to their partner's long-term fatigue:

Now, we have to forward plan how busy they will be and his fatigue and all these things you haven't thought about before. Like swimming. We went swimming once and he was just walking in the water but only ten minutes. He had to get out with me, with the two children, and then he had to sleep. (8: survivor)

For peer review only - http://bmjopen.bmj.com/site/about/guidelines.xhtml

Theme 3: Multiple losses and some gains

Participants grieved multiple losses, and sometimes cried alone or together (*I try my best not to cry in front of my daughter…. Other times I will cry to let her know that it is fine to cry. It's fine to let it out.* 3: survivor*).* For those whose partner died, these feelings were acute close to the event: *The day after … I had the most excruciating stomach pain myself. And I hoped it was AstraZeneca-related and that it would finish me off too: I felt incredibly lonely….* 3: bereaved*).*

Loss of a loved one

For those who were bereaved, the devastation was evident:

> *It just seems so cruel. It's the cruelty of it. And it's the fact that it's irreversible. There's nothing. It's a helplessness. That is the worst - there's nothing you can do to change this situation.* (5: bereaved)

> *I was on a lot of medication immediately following. I started having panic attacks, which I'd never had before. I was on antidepressants, sleeping tablets, and diazepam. I'd never taken anything before. I'd always been really down to earth, grounded… I couldn't remember anything. I don't know if it's a combination of the drugs or the trauma or what, but I just couldn't remember a thing. I was sleeping a maximum of two hours a night, which was not helping anything. I didn't dream much, thank God, but when I did, it wasn't nice. … Yeah, just lots of very irrational ways. I couldn't read… That was really torturous. I can't do anything. I can't think. I can't watch TV. I can't take anything in. I can't hold a conversation.* 2: bereaved

Family members of survivors mourned the loss of the person they used to know (*It's almost a grief that you deal with* 9: survivor). For those with continuing health problems, outcomes such as fatigue, difficulties in mobilisation, and low mood reduced not only the quality of life of the individual affected but also the quality of relationships with those around them.

> *He's not the husband that I had and he's not the dad that he's always been and wants to be, and he's not the granddad that he's always thought he would be. … I mean, it's been what, two years... But since he's been out of hospital, there's just been a slow decline…. And it's horrible to watch him just slipping away bit by bit…* (3: survivor)

> *Even now, the fatigue… She does an hour's work, and she's knackered. You don't see her before and after picture. I see the woman before that was sort of 8 stone, 9 stone, two kids,*

For peer review only - http://bmjopen.bmj.com/site/about/guidelines.xhtml

> *went out to work. Because I was out of work, she chose what she wanted to do so she'd do it.* 5: survivor

'Good days' are rare, but welcomed (…*you know, she knows like when he's on a good day, you know. She goes in and starts chatting away like she does. She always goes to him for the chats.* (5: survivor), while roles within the relationship change and may even be reversed.

Loss of a future

Another of the multiple losses these individuals experienced was the loss of an expected future:

> *There's been some very dark, dark days and nights as such, where I really needed him to be the X that I know - and he can't so yeah, I just carry on… You just get up, you get dressed, and you see where the day goes and do what you can. And then you start all over again the next day. …. I've never been wanting these massive holidays and do this do that, the nights out, you know. We've never really done all that, but just be able to just go out maybe for our anniversary … that would have been nice.* (3: survivor)

One participant whose partner died summed up the experience of many, including those whose partner survived with continuing symptoms:

> *We're thinking about slowing down. Enjoying your life now. You're getting older. In fact, X, he said, "After lockdown ends, we'll go on holiday. We'll do something. Children are grown up now. It's our time." So, it's all those things that are left with you, all those unfulfilled dreams, all those plans we had for the future.* (5: bereaved)

Another stated:

> *I'm still trying to do the things that give pleasure. But I realised the thing that gave me the most pleasure in my life was my marriage to my husband. And it feels like my world is never going to be the same because I don't have anyone in it who loves me like he did; nor do I have anyone to love in the way I loved him.* (3: bereaved)

More pragmatic losses of future financial security were also evident, with many relying on redundancy, universal credit, or PIP to support them in constrained financial circumstances. One family sold a business, at a considerably lower than optimal price. Others survived on redundancy money that was gradually reducing over time, with consequent increasing limitations of the quality of life and availability of valued experiences.

For peer review only - http://bmjopen.bmj.com/site/about/guidelines.xhtml

From trauma, growth

By contrast, a number of participants, particularly among the survivors, reported personal growth both as individuals and as a family, as a consequence of their experiences:

> *I never knew that I could be so patient and compassionate…* (6: survivor)

> *And I think having a near death experience possibly might have made me more positive about things, appreciating life a bit more..*(3: survivor)

> *It slowed us down and made us sit and think, there's more to life than this. There's kids, there's family. I mean, the family…. Some good comes out of some of it.* (5: survivor)

Theme 4: Battling against the system

One of the starkest issues reported by families of patients who died were 'battles' with the hospitals that cared, from their perspectives less than optimally, for their partner and the coroners involved in the death certification. Two made formal complaints about the hospital care. Three reported significant dissatisfaction with the process in the coroner's court, the medical representatives involved, or even their own legal team who failed to challenge some of the 'questionable' medical decisions and hospital processes (*…they weren't challenging the doctor who did the same-day referral. They weren't challenging…. So, for me, I just felt, (expletive deleted), you are an absolute waste of space:* (3: bereaved).

Three participants challenged death certificates which did not confirm VITT as cause of death, despite this being stated in medical notes: vital information, partly because partners wished the truth to be spoken about why their partner died, and more pragmatically because without a diagnosis of VITT on the death certificate they were not eligible for compensation under the Vaccine Damage Compensation Scheme (VDPS). Despite access to significant legal support, and in one case lobbying by both their GP and specialist nurse, participants found these challenges stressful and exhausting, an unwanted battle at a time they were grieving. As one person stated:

> *And by that point, when the coroner rang me to say he died of natural causes. After the anger has subsided, after a day or two, I rang the coroner's office. And I said, "I still don't agree with this. I want another inquest. I want..." And the chap … he kind of laughed at me. He said, "It can take you years and can cost you a lot of money. They won't agree to this."*

For peer review only - http://bmjopen.bmj.com/site/about/guidelines.xhtml

And there was not much more I could say, but I thought, "I'm not letting this go. That's for sure." (5: bereaved).

Another participant (3: bereaved) also noted: *... I need to complain about the coroners because nothing will change and I'm not convinced even if I do complain, anything will change. But if I don't, then it definitely won't.*

Even when the role of the vaccine was acknowledged by the coroner, this decision was not always immediate; and was only accessible after a struggle:

I felt like I had to harass them to get any information and that it was very limited.... We were just left wondering for 10 months. (1: bereaved)

Accessing the VDPS was initially very difficult and involved significant and excessive bureaucracy:

(*[VDPS} said, oh we need your husband's medical records. I said, don't be ridiculous. Why would you need his medical records? You've got a death certificate that says Astra Zeneca killed him. What more do you want? That's conclusive proof.* (3: bereaved*)*

And even death did not necessarily fulfil the required level of disability:

T*he initial contact with the [VDPS] was to explain the situation and ask whether I'd be eligible. And the reply was basically, even if you can establish causation, death does not necessarily satisfy the 60% disability criteria!* (1: bereaved)

Working through the VDPS was complex, and often ended in surviving patients failing to meet the criteria for funding, despite them being unable to work in either their previous or new employment, or receiving funding after considerable delays. This placed many in financial jeopardy. In addition, as noted above, even when patients had died, accessing the funding proved difficult.

For a number of participants, the need to publicise the risks associated with the vaccine was driven by anger at the lack of information at the time of vaccination:

So, I guess for a while I was just very angry that ...you know... he wasn't forced *to take it but then the kind of like realization that there wasn't any ... how can they not inform us properly, how can they not tell me? Like, you know I'm not medical, so when they're telling*

me 'Have a vaccine', why aren't they telling me that there could be side effects? So, I was very confused and very sort of angry about. (4: bereaved)

As a consequence, some became deeply politically and emotionally involved in attempts to publicise the potential side-effects of vaccination through Instagram and other social media: posts and groups which were frequently taken down as they were identified as antivax, which, collectively, they were not:

> *I was telling people to get vaccinated. I was just saying, "But be aware. If you have any side effects, don't do what we did. Don't be told, "Oh, it's a migraine, you'll be fine," because it's not. It could be something much more dangerous.* (2: bereaved)

Likewise, mainstream media reported little or anything of their experiences:

> *I remember talking to an ex-ITV reporter who had got me in contact with ITV and This Morning, the programme, the news programme, and they had invited me on to share my story. So that's okay, great you know. And then I got a phone call from that reporter that said due to Ofcom regulations we can't have any story that would negatively impact the uptake of the vaccine.* (4: bereaved)

So, publicity was largely achieved through multiple spots on GBNews, although this was viewed with some cynicism:

> *... It's like even the media outlets that give you a platform. It's always for a benefit of theirs you know. It's not really to help. It's to be alternative it's to be 'look what we're doing we're talking to these people'.* (4: bereaved)

As a consequence, many of the family members of both deceased and surviving VITT patients (and other vaccine damaged individuals) have formed a number of action groups, with the largest being VIBUK, in an attempt to influence the government and to instigate changes to the system, with support from one lawyer working *pro bono* on their behalf. Meetings have been held with parliamentarians, with some successes, although this brings its frustrations:

> *... when I watch Sir Christopher Choke talking in the House of Parliament and there's two people sitting there and neither of them are listening. They're on their phone. So, I feel I feel very you know, like they just don't want.. there's no respect at all. There's no like, you know, if anyone was listening to somebody who had lost someone in any way in any capacity, there would be a human element to respond with compassion.* 4: bereaved

For peer review only - http://bmjopen.bmj.com/site/about/guidelines.xhtml

DISCUSSION

This is the first study to report the psychosocial impact of the VITT beyond the directly affected individual. As always with qualitative studies, the sample was small and potentially biased through its sampling method. However, the number of bereaved participants accounted for approximately 10 per cent of all VITT-bereaved UK families.

Even with these provisos, the study captured a range of negative impacts on partners, children and parents of VITT patients. Many of the outcomes were inevitable and, whilst painful for those involved, could not be addressed by the health and legal systems. Others could. These include developing appropriate systems for supporting families during the in-patient period when direct access to clinicians and/or patients is not possible, preparation for very sick and disabled patients returning home, and attention to longer-term support for those with high caring burdens. Consideration also needs to be given to how both the coroners' courts and VDPS respond to people with significant needs and under significant stress, both emotional and financial. Finally, and echoing the findings in the previous study, the government needs to reflect on how to support and give voice to individuals and families in similar contexts in the future, should they arise.

Contributor statement:

Paul Bennett: interviews and lead in design, analysis and authorship of the study.

Filiz Celik: interviews, co-analysis, and contribution to authorship of the study.

Jenna Winstanley: interviews and contribution to authorship of the study

Beverley Hunt: contributions to design and authorship of the study

Sue Pavord: contributions to design and authorship of the study

Competing interests: No author has any competing interests in relation to this study.

Funding: The research reported here received no specific grant from any funding agency in the public, commercial or not-for-profit sectors

Data sharing statement: raw data (anonymised transcripts) are available from the corresponding author (PB)

Ethics approval: The study was given ethical approval by the Swansea University School of Psychology ethics committee (approval no. 5513-4722).

For peer review only - http://bmjopen.bmj.com/site/about/guidelines.xhtml

For peer review only

REFERENCES

1. Bennett P, Celik F, Winstanley J, *et al* Living with vaccine-induced immune thrombocytopenia and thrombosis: a qualitative study *BMJ Open* 2023/ bmjopen-2023-072658
2. Pavord S, Scully M, Hunt BJ, et al. Clinical features of Vaccine-Induced Immune Thrombocytopenia and Thrombosis. *N Engl J Med* 2021; 385:1680-1689.
3. Braun V, Clarke V. Using thematic analysis in psychology. *Qual Res Psychol* 2006; *3*: 77-101.

For peer review only

Topic guide

Record!

Check consent - aware that is being recorded, can drop out at any time, audio recordings will be destroyed and anonymized data used in research outputs.

Age

Gender

Relationship to VITT person

Want to discuss the impact of VITT on you and perhaps any others within the family affected by this… start with what has happened and then its impact on you/family

1. Describe the family
2. Thinking back to the time when partner/family member developed VITT, how did this impact on you and other members of the family?

   Prompts include…

For peer review only

- what experiences for you/them during initial onset of symptoms/hospitalization?
- When did you find symptoms were VITT?
- What about family? How broke the news, how took it?

3.What were the psychological consequences of these experiences at the time for you/them?

Prompts include..

- Impact of isolation due to covid
- Fear, worry

3. What physical/psychological symptoms has person with VITT since then? How have these impacted on your (joint) life?
   Prompts include…
   - Day to day – mobility, pain, fatigue restrictions
   - Longer term – finance, job
   - Impact on family

4. What were the longer-term psychological consequences of living with a person with VITT?

Prompts include

For peer review only

- Health anxiety
- Depression
- Concern for partner
- Anger/frustration
- Restricted family quality of life

5. If partner deceased.. what have been your experiences since your loss?
   Prompts include
   - Emotional
   - Contact with other bereaved people
   - The system.. hospital, VDPS etc
   - How impacted on family

6. *If time*, what are views on vaccination/covid now?

Thanks for participating. Reminder that if need support get in touch with Thrombosis UK on email in the PIS.

Will send copy of findings…

For peer review only

Standards for Reporting Qualitative Research (SRQR)*

http://www.equator-network.org/reporting-guidelines/srqr/

Title and abstract

	Page/line no(s).
**Title** - Concise description of the nature and topic of the study Identifying the study as qualitative or indicating the approach (e.g., ethnography, grounded theory) or data collection methods (e.g., interview, focus group) is recommended	P3
**Abstract** - Summary of key elements of the study using the abstract format of the intended publication; typically includes background, purpose, methods, results, and conclusions	P2

Introduction

**Problem formulation** - Description and significance of the problem/phenomenon studied; review of relevant theory and empirical work; problem statement	P3
**Purpose or research question** - Purpose of the study and specific objectives or questions	P3

Methods

**Qualitative approach and research paradigm** - Qualitative approach (e.g., ethnography, grounded theory, case study, phenomenology, narrative research) and guiding theory if appropriate; identifying the research paradigm (e.g., postpositivist, constructivist/ interpretivist) is also recommended; rationale**	P3-4
**Researcher characteristics and reflexivity** - Researchers' characteristics that may influence the research, including personal attributes, qualifications/experience, relationship with participants, assumptions, and/or presuppositions; potential or actual interaction between researchers' characteristics and the research questions, approach, methods, results, and/or transferability	P4
**Context** - Setting/site and salient contextual factors; rationale**	P3-4
**Sampling strategy** - How and why research participants, documents, or events were selected; criteria for deciding when no further sampling was necessary (e.g., sampling saturation); rationale**	P3
**Ethical issues pertaining to human subjects** - Documentation of approval by an appropriate ethics review board and participant consent, or explanation for lack thereof; other confidentiality and data security issues	P15
**Data collection methods** - Types of data collected; details of data collection procedures including (as appropriate) start and stop dates of data collection and analysis, iterative process, triangulation of sources/methods, and modification of procedures in response to evolving study findings; rationale**	P3-4

For peer review only - http://bmjopen.bmj.com/site/about/guidelines.xhtml

**Data collection instruments and technologies** - Description of instruments (e.g., interview guides, questionnaires) and devices (e.g., audio recorders) used for data collection; if/how the instrument(s) changed over the course of the study	P3 and uploaded topic guide
**Units of study** - Number and relevant characteristics of participants, documents, or events included in the study; level of participation (could be reported in results)	P3
**Data processing** - Methods for processing data prior to and during analysis, including transcription, data entry, data management and security, verification of data integrity, data coding, and anonymization/de-identification of excerpts	P4
**Data analysis** - Process by which inferences, themes, etc., were identified and developed, including the researchers involved in data analysis; usually references a specific paradigm or approach; rationale**	
**Techniques to enhance trustworthiness** - Techniques to enhance trustworthiness and credibility of data analysis (e.g., member checking, audit trail, triangulation); rationale**	P4

Results/findings

**Synthesis and interpretation** - Main findings (e.g., interpretations, inferences, and themes); might include development of a theory or model, or integration with prior research or theory	P4-13
**Links to empirical data** - Evidence (e.g., quotes, field notes, text excerpts, photographs) to substantiate analytic findings	P4-13

Discussion

**Integration with prior work, implications, transferability, and contribution(s) to the field -** Short summary of main findings; explanation of how findings and conclusions connect to, support, elaborate on, or challenge conclusions of earlier scholarship; discussion of scope of application/generalizability; identification of unique contribution(s) to scholarship in a discipline or field	P15
**Limitations** - Trustworthiness and limitations of findings	P15

Other

**Conflicts of interest** - Potential sources of influence or perceived influence on study conduct and conclusions; how these were managed	P15
**Funding** - Sources of funding and other support; role of funders in data collection, interpretation, and reporting	P15

*The authors created the SRQR by searching the literature to identify guidelines, reporting standards, and critical appraisal criteria for qualitative research; reviewing the reference lists of retrieved sources; and contacting experts to gain feedback. The SRQR aims to improve the transparency of all aspects of qualitative research by providing clear standards for reporting qualitative research.

For peer review only - http://bmjopen.bmj.com/site/about/guidelines.xhtml

**The rationale should briefly discuss the justification for choosing that theory, approach, method, or technique rather than other options available, the assumptions and limitations implicit in those choices, and how those choices influence study conclusions and transferability. As appropriate, the rationale for several items might be discussed together.

Reference:
O'Brien BC, Harris IB, Beckman TJ, Reed DA, Cook DA. **Standards for reporting qualitative research: a synthesis of recommendations.** *Academic Medicine*, Vol. 89, No. 9 / Sept 2014
DOI: 10.1097/ACM.0000000000000388

For peer review only

BMJ Open

A qualitative study of the consequences of vaccine-induced immune thrombocytopenia and thrombosis: the experiences of family members.

Journal:	*BMJ Open*
Manuscript ID	bmjopen-2023-080363.R1
Article Type:	Original research
Date Submitted by the Author:	17-Nov-2023
Complete List of Authors:	Bennett, Paul; Swansea University, Psychology<br>Celik, Filiz; Swansea University<br>Winstanley, Jenna; No affiliation<br>Hunt, Beverley; Kings Healthcare Partners, Thrombosis and Haemophilia Centre<br>Pavord, Sue; Oxford University Hospitals NHS Foundation Trust,
**Primary Subject Heading**:	Public health
Secondary Subject Heading:	Global health
Keywords:	Bleeding disorders & coagulopathies < HAEMATOLOGY, PUBLIC HEALTH, Vaccination, Quality of Life, QUALITATIVE RESEARCH

SCHOLARONE™
Manuscripts

I, the Submitting Author has the right to grant and does grant on behalf of all authors of the Work (as defined in the below author licence), an exclusive licence and/or a non-exclusive licence for contributions from authors who are: i) UK Crown employees; ii) where BMJ has agreed a CC-BY licence shall apply, and/or iii) in accordance with the terms applicable for US Federal Government officers or employees acting as part of their official duties; on a worldwide, perpetual, irrevocable, royalty-free basis to BMJ Publishing Group Ltd ("BMJ") its licensees and where the relevant Journal is co-owned by BMJ to the co-owners of the Journal, to publish the Work in this journal and any other BMJ products and to exploit all rights, as set out in our licence.

The Submitting Author accepts and understands that any supply made under these terms is made by BMJ to the Submitting Author unless you are acting as an employee on behalf of your employer or a postgraduate student of an affiliated institution which is paying any applicable article publishing charge ("APC") for Open Access articles. Where the Submitting Author wishes to make the Work available on an Open Access basis (and intends to pay the relevant APC), the terms of reuse of such Open Access shall be governed by a Creative Commons licence – details of these licences and which Creative Commons licence will apply to this Work are set out in our licence referred to above.

Other than as permitted in any relevant BMJ Author's Self Archiving Policies, I confirm this Work has not been accepted for publication elsewhere, is not being considered for publication elsewhere and does not duplicate material already published. I confirm all authors consent to publication of this Work and authorise the granting of this licence.

For peer review only

A qualitative study of the consequences of vaccine-induced immune thrombocytopenia and thrombosis: the experiences of family members.

Paul Bennett PhD (corresponding author)
Thrombosis UK and Swansea University
School of Psychology
Swansea University
Swansea
SA2 8PP
p.d.bennett@swansea.ac.uk

Filiz Celik PhD
School of Psychology
Swansea University
Swansea
UK
Filiz.celik@swansea.ac.uk

Jenna Winstanley BSc
No affiliation
jvwinstanley@outlook.com

Beverley J Hunt OBE
Thrombosis & Haemophilia Centre
Kings Healthcare Partners
London
Beverley.hunt@gstt.nhs.uk

Sue Pavord FRCPath
Department of Haematology
Oxford University Hospitals NHS FT
Sue.pavord@ouh.nhs.uk

For peer review only

ABSTRACT

Objectives: To explore the experiences of family members of patients who died or survived following a diagnosis of Vaccine-induced Immune Thrombocytopenia and Thrombosis (VITT).

Design: A semi-structured qualitative study, conducted via Zoom.

Setting: Participants discussed their experiences during hospitalisation and following discharge.

Participants: Sixteen family members of VITT patients (survivors = 11; bereaved = 5), recruited via a Facebook support group and advertising on Twitter.

Results: Analysis identified two themes common to both groups of participants: the stress of hospitalisation, and the experience of multiple losses. A third theme, living with VITT, was unique to the survivor group, and a fourth, battling against the system, was predominantly reported by bereaved participants.

Conclusions: This is a significantly challenged group of people, with multiple emotional, financial, social and psychological losses. These losses have been compounded by experiences of limited governmental and societal recognition of the problems they face.

Key words: family, vaccine-induced immune thrombocytopenia and thrombosis, VITT, psychosocial sequelae

STRENGTHS AND LIMITATIONS OF THIS STUDY

Strengths

- The first study to explore the impact on the families of patients surviving and dying of VITT
- The sample included approximately 10 per cent of the surviving relatives of all UK patients who died from VITT.

Limitations

- Although attempts were made to widen recruitment, the majority were from a VITT support group provided by Thrombosis UK and in response to tweets via Twitter (X)

Word count: 4823

For peer review only

INTRODUCTION

We have previously explored the experiences of patients diagnosed with Vaccine-induced Immune Thrombocytopenia and Thrombosis (VITT) over the 18 months since their acute presentation [1]. During these interviews, it became apparent that the experiences of patients' families (including health, financial, and social concerns) largely mirrored those of the patients, although this was not formally explored in the study. Furthermore, a mortality rate of more than 20 per cent [2] necessarily meant that some families had to cope with the sudden and unexpected death of a family member, often at a young age. The present study aimed to explore the experiences of both bereaved and survivor families.

METHOD

Qualitative interviews were conducted 15-24 months following a diagnosis of VITT.

Patient and public involvement

The study protocol was discussed separately with several relatives of patients with VITT prior to conducting the research. In addition, all participants had repeated sight of the paper as it developed to ensure their agreement with the themes identified, quotations cited, and degree of anonymity provided.

Recruitment and sample

Participants were family members of patients with a confirmed diagnosis of VITT, recruited via social media: (i) a closed VITT Facebook support group hosted by Thrombosis UK, (ii) the Thrombosis UK Twitter feed, and (iii) re-tweets by individuals known to members of the Facebook group or who read the Twitter feed. The final sample comprised 16 participants. Eleven were family members of surviving VITT patients (nine partners, two adult daughters). Five were bereaved. There were 13 women and 3 men, with a median age of 53 years (range 21-59).

Method

Interviews were conducted between August 2022 and July 2023. People who contacted the study team were sent a Participant Information Sheet and confirmed their willingness to participate by making further contact with the study lead. After providing informed consent, participants took part in a semi-structured virtual interview on Zoom, addressing their experiences of hospitalisation and the longer-term impact of living with a VITT patient or of

For peer review only

their bereavement (see appendix 1). Three people (PB, FC, JW,) trained in clinical/counselling psychology and with previous experience of VITT [1], conducted the interviews.

Data Analysis

Interviews were recorded on Zoom and a 'clean' transcript determined, after which the original recordings were deleted. All identifying elements were removed from the transcripts, which were analysed by PB using inductive thematic analysis [3]. Audio recordings were listened to repeatedly to identify relevant initial codes and textual units. Extracts and phrases were used to identify potential themes, with relevant quotes gathered within identified themes. Themes and sub-themes derived from the data were checked and validated by FC; any differences were resolved through discussion.

RESULTS

Of the themes identified, theme 1, the stress of hospitalisation, has commonalities across both groups of participants. Theme 2, Living with VITT, is unique to families of survivors. Theme 3, multiple losses, has commonalities across both groups. Theme 4, battling against the system, is predominantly reported by bereaved participants. Table 1 provides an overview of the themes and sub-themes.

Table 1: Key themes and sub-themes

Theme	Sub-theme
1. The stress of hospitalisation	The dawning light of seriousness
	Breaking the bad news
	Waiting for news
2. Living with VITT	Coming home is not the end
	The whole family has to adjust
3. Multiple losses and some gains	Loss of a loved one
	Loss of a future
	From trauma, growth
4. Battling against the system	

Theme 1: the stress of hospitalisation

For peer review only - http://bmjopen.bmj.com/site/about/guidelines.xhtml

The dawning light of seriousness

Most participants were concerned at the initial onset of symptoms. However, this anxiety increased significantly when participants became aware of the severity of their family member's diagnosis, often given over the phone due to Covid pandemic imposed visiting limitations. Anxiety was frequently exacerbated by incomplete understandings of the medical condition:

> *I had a consultant phone, and she was saying, ... She's reading through a big, long list of things. .... She's going down this list. And then she says, "Oh, and she's had a stroke." That's the one word that my mind picked up on because that's what I knew about. ... She went, "Yes, but don't worry about that. That's the minor thing what's wrong with her." That's when you think, God ... The stroke is the minor thing! ... what's the rest of it!? (*5: survivor*)*

Many relatives of survivors were fearful they would die. As one participant noted, '*I suddenly woke up, like bang at four o'clock in the morning, and I thought... she could actually be dead now. She could be dead now.'* (7: survivor). For those who did die, there was little or no time for preparation. Four of the five participants were rung at home shortly after admission, told that exploratory surgery have proven unsuccessful, and that their partner had very limited time to live:

> *I remember crying a hell of a lot. But because it was just so quick... I was more shocked. I remember physically feeling sick to my stomach, and then it just sort of clicked... scared sick or something or something like that because I was literally physically sick. I just remember feeling really raw and empty, and just shocked.* (4: bereaved)

> *And I was with him then. His parents rang my mobile while I was with him... and they heard me tell him that they loved him... And then they hung up.* (3: bereaved).

Children's worries about their parent's health manifested in several ways. One parent (5: survivor) had to sleep with their child to help keep them calm. One child resorted to prayer (*You know how children pray by the bedside... and she was going. Daddy, please don't die, please don't die* 6: survivor*)*, while others acted out their stress at school (*A teacher contacted me and said, "Your son's been in a bit of a mess in the class because he's worried about his mom".* 2: survivor).

For some family members, trauma continued in the form of flashbacks or memories of critical events for many months: *I think now it's hitting me. Because I'm getting flashbacks and things like that. ... And my mind's trying to deal with it. ... And just sort a lying there, just shaking and not*

For peer review only - http://bmjopen.bmj.com/site/about/guidelines.xhtml

knowing who I am or where I am; and I'm not recognizing my room and not knowing my name. (1: survivor).

Breaking the bad news

In the face of their own concerns, participants had to determine when and how to inform others of their partner's condition. Telling children was perhaps even more challenging. Participants had to impart that their parent was sufficiently ill to require hospitalisation, but try to maintain optimism and confidence (despite their own reservations) that they would return home:

> *I was thinking, what do I say? Do I tell them? I thought it was probably best not to at this stage because they will not sleep… it'll just make them in a bit of a mess…. I was like, "Oh, well, she's staying in overnight, and everything will be all right, let's get to bed." The other thing I thought about it was, what if I'm telling the kids, "Everything's all right, everything's all right.", then in the morning, I'm going to get this message saying, your wife had a stroke or died in the night, and then I'm going to tell them this and actually she wasn't all right, and I was lying to them.* (2: survivor)

Waiting for news

Following admission to hospital, anxieties were often exacerbated by Covid-related visiting restrictions which resulted in very limited contact with clinicians. Relatives knew their condition was serious, but most struggled to track how their relative was progressing:

> *.. The biggest thing was not being with him. That was the hardest thing, and just waiting for... Because the hospital, they never rang me. The nurses, the doctors never rang me to say, "This is what's happened."* (5: bereaved)

When achieved, communication could also be challenging and distressing for both participants and health professionals:

> *… because they took all of the hope away from me. One particular doctor told me over the phone that all of the interventions they were doing, pretty much, were a waste of time and never called me back to talk me through that because I had to put the phone down.* (8: survivor)

Communication with patients often involved the use of social media, which could be reassuring, but could also be stressful:

For peer review only

I just laid here just waiting for the phone to go. Just waiting for a message saying, 'I'm fine', you know. He was losing all track of time and he'd send me messages at like 4:00 o'clock in the morning… It was just horrendous. All the things that go through your head. Just staring at the four walls and. Yeah, just waiting. (3: survivor)

The lack of direct contact with clinicians and patients led to anxieties about the veracity of patient reports about their health:

I think there was always a worry that my mum was not going to be entirely 100% honest with it, not out of anything other than just like trying not to worry me. I found it hard… (7: survivor)

Finally, participants worried about the psychological impact of hospitalisation on the patient as well as experiencing their own stress:

Whenever she's been ill or anything like that, I've always been with her. Not to be with her is quite upsetting because I'm then thinking of how scared and upset she's going to be… I came home and literally stood in the garden and cried. That's being military, 23 years in the army, quite hands-on, quite a person to get things done and fix things and make things right. It's horrible not being in control. (9: survivor)

Theme 2: Living with VITT

Coming home is not the end

For survivors, initial relief following discharge was frequently replaced by the significant challenges of living with, and caring for, a very sick individual, who may have changed significantly from the person before admission to hospital.

And the mood swings. You know, I think it's 'cos it's coming down off all the steroids and everything else. The mood swings that he had was... awful … just horrendous. He wasn't aggressive, but he just didn't realise how snappy he was getting…You just, you didn't know whether to speak, not speak. Do you go in? Do you not? You know, it was horrible. I remember my girl crying herself to sleep in my arms because it wasn't her dad that came home. (3: survivor)

For peer review only

Participants were concerned about the risk of future problems, and how to avoid them. They expressed concerns about their partner's health as well as awareness that medical aid was not immediately available:

> *Yeah. I suppose when she came home, I was treating her with kid gloves. I felt like she was a bit, she was super-delicate, and I kept thinking don't bang your head, don't do anything when you knock your head or... And that probably got on her nerves a bit...* (2: survivor)

For some, recovery from VITT meant a slow acceptance of improving health, confidence that they will not experience further health crises, and shifts towards a more normal life. For those nine individuals with continuing symptoms, the stresses and strains continued.

> *I lie there at night listening to his breathing 'cos .... he struggles a lot in the night. And some nights I nudge him to make sure he's going to take the next breath, cause he's really struggling to breathe, so I know if in the night his breathing is real bad I know in the morning, what kind of day it's gonna be.* (3: survivor)

As well as the daily demands of caring, participants noted the toll of repeated hospital appointments (one participant noted their partner's 250th appointment had recently passed) either for monitoring, rehabilitation, or continuing investigations. Others family members reported the sheer stress of caring:

> *.... in the early days, it was a massively dark time in the fact that she was so angry and upset. She didn't want to be here anymore. Trying to keep her going, trying to keep her functioning, trying to get the right pills in her, trying to work out what's going on and trying to work out what appointment's coming next. ... But we still have other days where it's dark and she gets angry and upset and she doesn't want to be here anymore. So, dealing with that is hard work...* (9: survivor)

> *I went through a really tired stage where I had this like chronic fatigue and I just, and it would just hit me wherever I was, and I'd actually have to go and lie down no matter what time of day it was. So that's exhaustion and fatigue*. (1: survivor)

Most families reported little or no systematic support following discharge (although some individual clinicians were highly supportive) even when the patient had experienced significant life-changing conditions:

For peer review only - http://bmjopen.bmj.com/site/about/guidelines.xhtml

..but there seems to be no sort of holistic approach to supporting families when a loved one has been through a life-changing condition in the hospital or in a coma. There's no pathway for that to support us. They just look at the patient… (8: survivor)

The whole family has to adjust

The impact of the illness frequently affected the whole family. For some, as noted above, this involved daily commitments to care on a 24-hours a day basis. For others, the commitment may have been less, but life plans were nevertheless changed.

My husband's job is in London, and we were thinking of moving up towards London. And after this happened, I decided let's buy a house here in X so I'm just three minutes away from my mum. And then my brother bought his and he's two minutes away from me. So, we're all literally in a line. So, we're all around to be here for her. (11: survivor)

At times, more subtle issues affected the whole family. Two participants, for example, reported the need to adjust the family day to day noise, and for their partner to have periods of quiet:

… we can't behave the way we used to. Like before, we'd carry on as a normal day to day family messing around, noise, you know, you'd have all that … And now it's like you're kind of walking on egg shells all the time because you don't know…. (6: survivor)

Another reported the adjustments necessary due to their partner's long-term fatigue:

Now, we have to forward plan how busy they will be and his fatigue and all these things you haven't thought about before. Like swimming. We went swimming once and he was just walking in the water but only ten minutes. He had to get out with me, with the two children, and then he had to sleep. (8: survivor)

Theme 3: Multiple losses and some gains

Participants grieved multiple losses, and sometimes cried alone or together (*I try my best not to cry in front of my daughter…. Other times I will cry to let her know that it is fine to cry. It's fine to let it out.* 3: survivor*)*. For those whose partner died, these feelings were acute close to the event: *The day after … I had the most excruciating stomach pain myself. And I hoped it was AstraZeneca-related and that it would finish me off too: I felt incredibly lonely….* 3: bereaved*)*.

Loss of a loved one

For peer review only - http://bmjopen.bmj.com/site/about/guidelines.xhtml

For those who were bereaved, the devastation was evident:

> *It just seems so cruel. It's the cruelty of it. And it's the fact that it's irreversible. There's nothing. It's a helplessness. That is the worst - there's nothing you can do to change this situation.* (5: bereaved)

> *I was on a lot of medication immediately following. I started having panic attacks, which I'd never had before. I was on antidepressants, sleeping tablets, and diazepam. I'd never taken anything before. I'd always been really down to earth, grounded… I couldn't remember anything. I don't know if it's a combination of the drugs or the trauma or what, but I just couldn't remember a thing. I was sleeping a maximum of two hours a night, which was not helping anything. I didn't dream much, thank God, but when I did, it wasn't nice. … Yeah, just lots of very irrational ways. I couldn't read… That was really torturous. I can't do anything. I can't think. I can't watch TV. I can't take anything in. I can't hold a conversation.* 2: bereaved

Family members of survivors mourned the loss of the person they used to know (*It's almost a grief that you deal with* 9: survivor). For those with continuing health problems, outcomes such as fatigue, difficulties in mobilisation, and low mood reduced not only the quality of life of the individual affected but also the quality of relationships with those around them.

> *He's not the husband that I had and he's not the dad that he's always been and wants to be, and he's not the granddad that he's always thought he would be. … I mean, it's been what, two years... But since he's been out of hospital, there's just been a slow decline…. And it's horrible to watch him just slipping away bit by bit…* (3: survivor)

> *Even now, the fatigue… She does an hour's work, and she's knackered. You don't see her before and after picture. I see the woman before that was sort of 8 stone, 9 stone, two kids, went out to work. Because I was out of work, she chose what she wanted to do so she'd do it.* 5: survivor

'Good days' are rare, but welcomed (*…you know, she knows like when he's on a good day, you know. She goes in and starts chatting away like she does. She always goes to him for the chats.* (5: survivor), while roles within the relationship change and may even be reversed.

Loss of a future

Another of the multiple losses these individuals experienced was the loss of an expected future:

> *There's been some very dark, dark days and nights as such, where I really needed him to be the X that I know - and he can't so yeah, I just carry on... You just get up, you get dressed, and you see where the day goes and do what you can. And then you start all over again the next day. .... I've never been wanting these massive holidays and do this do that, the nights out, you know. We've never really done all that, but just be able to just go out maybe for our anniversary ... that would have been nice.* (3: survivor)

One participant whose partner died summed up the experience of many, including those whose partner survived with continuing symptoms:

> *We're thinking about slowing down. Enjoying your life now. You're getting older. In fact, X, he said, "After lockdown ends, we'll go on holiday. We'll do something. Children are grown up now. It's our time." So, it's all those things that are left with you, all those unfulfilled dreams, all those plans we had for the future.* (5: bereaved)

Another stated:

> *I'm still trying to do the things that give pleasure. But I realised the thing that gave me the most pleasure in my life was my marriage to my husband. And it feels like my world is never going to be the same because I don't have anyone in it who loves me like he did; nor do I have anyone to love in the way I loved him.* (3: bereaved)

More pragmatic losses of future financial security were also evident, with many relying on redundancy, universal credit, or PIP to support them in constrained financial circumstances. One family sold a business, at a considerably lower than optimal price. Others survived on redundancy money that was gradually reducing over time, with consequent increasing limitations of the quality of life and availability of valued experiences.

From trauma, growth

By contrast, a number of participants, particularly among the survivors, reported personal growth both as individuals and as a family, as a consequence of their experiences:

> *I never knew that I could be so patient and compassionate...* (6: survivor)

> *And I think having a near death experience possibly might have made me more positive about things, appreciating life a bit more..*(3: survivor)

For peer review only - http://bmjopen.bmj.com/site/about/guidelines.xhtml

> *It slowed us down and made us sit and think, there's more to life than this. There's kids, there's family. I mean, the family.... Some good comes out of some of it.* (5: survivor)

Theme 4: Battling against the system

One of the starkest issues reported by families of patients who died were 'battles' with the hospitals that cared, from their perspectives less than optimally, for their partner and the coroners involved in the death certification. Two made formal complaints about the hospital care. Three reported significant dissatisfaction with the process in the coroner's court, the medical representatives involved, or even their own legal team who failed to challenge some of the 'questionable' medical decisions and hospital processes (*…they weren't challenging the doctor who did the same-day referral. They weren't challenging…. So, for me, I just felt, (expletive deleted), you are an absolute waste of space:* (3: bereaved).

Three participants challenged death certificates which did not confirm VITT as cause of death, despite this being stated in medical notes: vital information, partly because partners wished the truth to be spoken about why their partner died, and more pragmatically because without a diagnosis of VITT on the death certificate they were not eligible for compensation under the Vaccine Damage Compensation Scheme (VDPS). Despite access to significant legal support, and in one case lobbying by both their GP and specialist nurse, participants found these challenges stressful and exhausting, an unwanted battle at a time they were grieving. As one person stated:

> *And by that point, when the coroner rang me to say he died of natural causes. After the anger has subsided, after a day or two, I rang the coroner's office. And I said, "I still don't agree with this. I want another inquest. I want..." And the chap ... he kind of laughed at me. He said, "It can take you years and can cost you a lot of money. They won't agree to this." And there was not much more I could say, but I thought, "I'm not letting this go. That's for sure."* (5: bereaved).

Another participant (3: bereaved) also noted: *… I need to complain about the coroners because nothing will change and I'm not convinced even if I do complain, anything will change. But if I don't, then it definitely won't.*

Even when the role of the vaccine was acknowledged by the coroner, this decision was not always immediate; and was only accessible after a struggle:

For peer review only - http://bmjopen.bmj.com/site/about/guidelines.xhtml

I felt like I had to harass them to get any information and that it was very limited.... We were just left wondering for 10 months. (1: bereaved)

Accessing the VDPS was initially very difficult and involved significant and excessive bureaucracy:

([VDPS} said, oh we need your husband's medical records. I said, don't be ridiculous. Why would you need his medical records? You've got a death certificate that says Astra Zeneca killed him. What more do you want? That's conclusive proof. (3: bereaved*)*

And even death did not necessarily fulfil the required level of disability:

The initial contact with the [VDPS] was to explain the situation and ask whether I'd be eligible. And the reply was basically, even if you can establish causation, death does not necessarily satisfy the 60% disability criteria! (1: bereaved)

Working through the VDPS was complex, and often ended in surviving patients failing to meet the criteria for funding, despite them being unable to work in either their previous or new employment, or receiving funding after considerable delays. This placed many in financial jeopardy. In addition, as noted above, even when patients had died, accessing the funding proved difficult.

For a number of participants, the need to publicise the risks associated with the vaccine was driven by anger at the lack of information at the time of vaccination:

So, I guess for a while I was just very angry that ...you know... he wasn't forced *to take it but then the kind of like realization that there wasn't any ... how can they not inform us properly, how can they not tell me? Like, you know I'm not medical, so when they're telling me 'Have a vaccine', why aren't they telling me that there could be side effects? So, I was very confused and very sort of angry about.* (4: bereaved)

As a consequence, some became deeply politically and emotionally involved in attempts to publicise the potential side-effects of vaccination through Instagram and other social media: posts and groups which were frequently taken down as they were identified as antivax, which, collectively, they were not:

For peer review only - http://bmjopen.bmj.com/site/about/guidelines.xhtml

I was telling people to get vaccinated. I was just saying, "But be aware. If you have any side effects, don't do what we did. Don't be told, "Oh, it's a migraine, you'll be fine," because it's not. It could be something much more dangerous. (2: bereaved)

Likewise, mainstream media reported little or anything of their experiences:

I remember talking to an ex-ITV reporter who had got me in contact with ITV and This Morning, the programme, the news programme, and they had invited me on to share my story. So that's okay, great you know. And then I got a phone call from that reporter that said due to Ofcom regulations we can't have any story that would negatively impact the uptake of the vaccine. (4: bereaved)

So, publicity was largely achieved through multiple spots on GBNews, although this was viewed with some cynicism:

... It's like even the media outlets that give you a platform. It's always for a benefit of theirs you know. It's not really to help. It's to be alternative it's to be 'look what we're doing we're talking to these people'. (4: bereaved)

As a consequence, many of the family members of both deceased and surviving VITT patients (and other vaccine damaged individuals) have formed a number of action groups, with the largest being VIBUK, in an attempt to influence the government and to instigate changes to the system, with support from one lawyer working *pro bono* on their behalf. Meetings have been held with parliamentarians, with some successes, although this brings its frustrations:

... when I watch Sir Christopher Choke talking in the House of Parliament and there's two people sitting there and neither of them are listening. They're on their phone. So, I feel I feel very you know, like they just don't want.. there's no respect at all. There's no like, you know, if anyone was listening to somebody who had lost someone in any way in any capacity, there would be a human element to respond with compassion. 4: bereaved

DISCUSSION

This is the first study to report the psychosocial impact of the VITT beyond the directly affected individual. As always with qualitative studies, the sample was small and potentially biased through its sampling method, which was, initially at least, based on the Thrombosis UK twitter feed and closed membership of its VITT patient group. That said, the number of bereaved participants accounted for approximately 10 per cent of all VITT-bereaved UK families.

For peer review only - http://bmjopen.bmj.com/site/about/guidelines.xhtml

The study captured a range of negative impacts on family members from both VITT and the acute exacerbation of distress as a consequence of the wider covid-related hospital restrictions. Many of the outcomes were inevitable, and, whilst painful for those involved, could not be addressed by the health and legal systems. Others could. These include developing appropriate systems for supporting families during the in-patient period when direct access to clinicians and/or patients is not possible, preparation for very sick and disabled patients returning home, and attention to longer-term support for those with high caring burdens. Consideration also needs to be given to how both the coroners' courts and VDPS respond to people with significant needs and under significant stress, both emotional and financial. Finally, and echoing the findings in the previous study, the government needs to reflect on how to support and give voice to individuals and families in similar contexts in the future, should they arise.

Contributor statement:

Paul Bennett was responsible for interviews and was lead in design, analysis and authorship of the study.

Filiz Celik conducted interviews, co-analysis, and contributed to authorship of the study.

Jenna Winstanley conducted interviews and contributed to authorship of the study.

Beverley Hunt contributed to the design and authorship of the study.

Sue Pavord contributed to the design and authorship of the study.

Competing interests: No author has any competing interests in relation to this study.

Funding: The research reported here received no specific grant from any funding agency in the public, commercial or not-for-profit sectors

Data sharing statement: raw data (anonymised transcripts) are available from the corresponding author (PB)

Ethics approval: The study was given ethical approval by the Swansea University School of Psychology ethics committee (approval no. 5513-4722).

REFERENCES

For peer review only

1. Bennett P, Celik F, Winstanley J, *et al* Living with vaccine-induced immune thrombocytopenia and thrombosis: a qualitative study *BMJ Open* 2023/ bmjopen-2023-072658
2. Pavord S, Scully M, Hunt BJ, et al. Clinical features of Vaccine-Induced Immune Thrombocytopenia and Thrombosis. *N Engl J Med* 2021; 385:1680-1689.
3. Braun V, Clarke V. Using thematic analysis in psychology. *Qual Res Psychol* 2006; *3*: 77-101.

For peer review only

Appendix 1:

Topic guide

Record!

Check consent - aware that is being recorded, can drop out at any time, audio recordings will be destroyed and anonymized data used in research outputs.

Age

Gender

Relationship to VITT person

Want to discuss the impact of VITT on you and perhaps any others within the family affected by this… start with what has happened and then its impact on you/family

1. Describe the family

For peer review only

2. Thinking back to the time when partner/family member developed VITT, how did this impact on you and other members of the family?

Prompts include…

- what experiences for you/them during initial onset of symptoms/hospitalization?
- When did you find symptoms were VITT?
- What about family? How broke the news, how took it?

3.What were the psychological consequences of these experiences at the time for you/them?

Prompts include..

- Impact of isolation due to covid
- Fear, worry

3. What physical/psychological symptoms has person with VITT since then? How have these impacted on your (joint) life?

Prompts include…

- Day to day – mobility, pain, fatigue restrictions
- Longer term – finance, job
- Impact on family

For peer review only

4. What were the longer-term psychological consequences of living with a person with VITT?

Prompts include

- Health anxiety
- Depression
- Concern for partner
- Anger/frustration
- Restricted family quality of life

5. If partner deceased.. what have been your experiences since your loss?

Prompts include

- Emotional
- Contact with other bereaved people
- The system.. hospital, VDPS etc
- How impacted on family

6. *If time*, what are views on vaccination/covid now?

Thanks for participating. Reminder that if need support get in touch with Thrombosis UK on email in the PIS.

For peer review only

Will send copy of findings…

For peer review only

Standards for Reporting Qualitative Research (SRQR)*

http://www.equator-network.org/reporting-guidelines/srqr/

Title and abstract

	Page/line no(s).
**Title** - Concise description of the nature and topic of the study Identifying the study as qualitative or indicating the approach (e.g., ethnography, grounded theory) or data collection methods (e.g., interview, focus group) is recommended	P3
**Abstract** - Summary of key elements of the study using the abstract format of the intended publication; typically includes background, purpose, methods, results, and conclusions	P2

Introduction

**Problem formulation** - Description and significance of the problem/phenomenon studied; review of relevant theory and empirical work; problem statement	P3
**Purpose or research question** - Purpose of the study and specific objectives or questions	P3

Methods

**Qualitative approach and research paradigm** - Qualitative approach (e.g., ethnography, grounded theory, case study, phenomenology, narrative research) and guiding theory if appropriate; identifying the research paradigm (e.g., postpositivist, constructivist/ interpretivist) is also recommended; rationale**	P3-4
**Researcher characteristics and reflexivity** - Researchers' characteristics that may influence the research, including personal attributes, qualifications/experience, relationship with participants, assumptions, and/or presuppositions; potential or actual interaction between researchers' characteristics and the research questions, approach, methods, results, and/or transferability	P4
**Context** - Setting/site and salient contextual factors; rationale**	P3-4
**Sampling strategy** - How and why research participants, documents, or events were selected; criteria for deciding when no further sampling was necessary (e.g., sampling saturation); rationale**	P3
**Ethical issues pertaining to human subjects** - Documentation of approval by an appropriate ethics review board and participant consent, or explanation for lack thereof; other confidentiality and data security issues	P15
**Data collection methods** - Types of data collected; details of data collection procedures including (as appropriate) start and stop dates of data collection and analysis, iterative process, triangulation of sources/methods, and modification of procedures in response to evolving study findings; rationale**	P3-4

For peer review only - http://bmjopen.bmj.com/site/about/guidelines.xhtml

**Data collection instruments and technologies** - Description of instruments (e.g., interview guides, questionnaires) and devices (e.g., audio recorders) used for data collection; if/how the instrument(s) changed over the course of the study	P3 and uploaded topic guide
**Units of study** - Number and relevant characteristics of participants, documents, or events included in the study; level of participation (could be reported in results)	P3
**Data processing** - Methods for processing data prior to and during analysis, including transcription, data entry, data management and security, verification of data integrity, data coding, and anonymization/de-identification of excerpts	P4
**Data analysis** - Process by which inferences, themes, etc., were identified and developed, including the researchers involved in data analysis; usually references a specific paradigm or approach; rationale**	
**Techniques to enhance trustworthiness** - Techniques to enhance trustworthiness and credibility of data analysis (e.g., member checking, audit trail, triangulation); rationale**	P4

Results/findings

**Synthesis and interpretation** - Main findings (e.g., interpretations, inferences, and themes); might include development of a theory or model, or integration with prior research or theory	P4-13
**Links to empirical data** - Evidence (e.g., quotes, field notes, text excerpts, photographs) to substantiate analytic findings	P4-13

Discussion

**Integration with prior work, implications, transferability, and contribution(s) to the field -** Short summary of main findings; explanation of how findings and conclusions connect to, support, elaborate on, or challenge conclusions of earlier scholarship; discussion of scope of application/generalizability; identification of unique contribution(s) to scholarship in a discipline or field	P15
**Limitations** - Trustworthiness and limitations of findings	P15

Other

**Conflicts of interest** - Potential sources of influence or perceived influence on study conduct and conclusions; how these were managed	P15
**Funding** - Sources of funding and other support; role of funders in data collection, interpretation, and reporting	P15

*The authors created the SRQR by searching the literature to identify guidelines, reporting standards, and critical appraisal criteria for qualitative research; reviewing the reference lists of retrieved sources; and contacting experts to gain feedback. The SRQR aims to improve the transparency of all aspects of qualitative research by providing clear standards for reporting qualitative research.

For peer review only

**The rationale should briefly discuss the justification for choosing that theory, approach, method, or technique rather than other options available, the assumptions and limitations implicit in those choices, and how those choices influence study conclusions and transferability. As appropriate, the rationale for several items might be discussed together.

Reference:

O'Brien BC, Harris IB, Beckman TJ, Reed DA, Cook DA. **Standards for reporting qualitative research: a synthesis of recommendations.** *Academic Medicine*, Vol. 89, No. 9 / Sept 2014 DOI: 10.1097/ACM.0000000000000388

For peer review only

For peer review only - http://bmjopen.bmj.com/site/about/guidelines.xhtml
